# The Neurological Impact of Leprosy: Manifestations and Treatment Approaches

**DOI:** 10.3390/neurolint16060111

**Published:** 2024-11-16

**Authors:** Andrea Calderone, Maria Catena Aloisi, Carmela Casella, Salvatore Fiannacca, Bruno Cosenza, Angelo Quartarone, Rocco Salvatore Calabrò

**Affiliations:** 1 Department of Clinical and Experimental Medicine, University of Messina, Piazza Pugliatti, 1, 98122 Messina, Italy; 2 IRCCS Centro Neurolesi Bonino-Pulejo, S.S. 113 Via Palermo, C.da Casazza, 98124 Messina, Italy; mariacatena.aloisi@irccsme.it (M.C.A.); salvatore.fiannacca@irccsme.it (S.F.); bruno.cosenza@irccsme.it (B.C.); angelo.quartarone@irccsme.it (A.Q.); roccos.calabro@irccsme.it (R.S.C.); 3 Stroke Unit, University of Messina, Piazza Pugliatti, 1, 98122 Messina, Italy; ccasella@unime.it

**Keywords:** leprosy, neurological complications, neuropathy, treatments

## Abstract

Background and Objectives: Leprosy primarily affects peripheral nerves, leading to significant neurological complications such as polyneuritis, mononeurosis, and autonomic dysfunction, which contribute to severe disabilities and impaired quality of life for patients. This scoping review aims to investigate the neurological manifestations and main treatments of leprosy patients. Materials and Methods: Studies were identified from an online search of PubMed, Web of Science, Cochrane Library, Embase, and Scopus databases. This review has been registered on OSF (n) PQBYH. Results: Neurological complications of leprosy, such as neuropathy and paralysis, necessitate accurate diagnosis and treatment, as immunological reactions can exacerbate nerve damage. Various studies highlight the effectiveness of personalized therapies, such as corticosteroids, multi-drug therapy (MDT), and surgical interventions, in improving symptoms and neurological function in leprosy patients. Conclusions: Managing neurological complications of leprosy necessitates careful diagnosis and treatment, as many patients experience unresolved peripheral neuropathy despite multidrug therapy. Future research should focus on improving diagnostic tools, exploring the link between neuropathic pain and psychological issues, and developing effective vaccines and treatments to enhance patient outcomes.

## 1. Introduction

**Figure 1:**
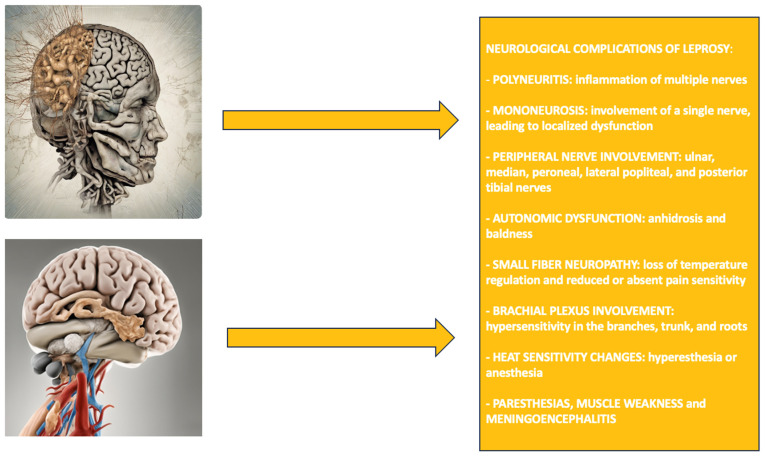
Neurological complications of leprosy.

Leprosy is a specific infectious disease with a long incubation period and a preference for the skin and nerves. Primary infection and immunological reversal cause nerve involvement, resulting in neurological dysfunction and severe disability [1]. Today, there are still countries with high rates of morbidity and disability, many of which are associated with nerve damage [2,3,4]. The countries with the highest number of new infections are India, Brazil, Indonesia, Bangladesh, and the Democratic Republic of the Congo. The incidence is higher in adults than in children, as more males are diagnosed (64% of cases) [5,6]. Mycobacterium leprae, an intracellular bacterium, infects skin, nerves, eyes, and joints. The primary replication is performed in Schwann cells, endothelial cells, and macrophages. Because it cannot be cultured in vitro, the mechanism of infection is hard to study, and thus, an incubation period can range from 3 to 10 years [7,8,9,10]. Leprosy is classified based on the Ridley–Jopling system and that of the World Health Organization (WHO). Ridley–Jopling categorizes it into five forms, which are tuberculoid leprosy, borderline tuberculoid, borderline, borderline lepromatous, and lepromatous leprosy. These classifications differ because of changes that vary in skin lesions and the form of the immune response. WHO classifies leprosy into mainly two groups: the first category includes only 1–5 lesions, no bacteria detected, called paucibacillary leprosy; the second category is multibacillary leprosy: more than 5 lesions with bacteria present. It is considered a multistem system disease. Skin, peripheral nerves, and the reticuloendothelial system are the main systems involved. Ocular involvement occurs in 70–75% of patients and 5% of blindness [11,12,13,14,15,16]. Nasal and oral mucosal lesions produce nasal blockage and nose deformity. The ocular manifestations are due to nerve damage and bacillary invasion. Skin lesions predominantly occur on cooler parts of the body, and hence, neurological manifestations are present, and peripheral nerves are involved [17,18,19]. The most common neurological form is polyneuritis, followed by mononeurosis, with heat acting as a factor in the development of nerve lesions [20,21,22]. Nerves that are essentially cold, such as the ulnar, median, peroneal, lateral popliteal, and posterior tibial nerves, are commonly involved, while the temporal pole is found to have increased sensitivity due to the auricular nerve [23,24,25]. Anhidrosis and baldness may be seen as autonomic dysfunction in leprosy patients, although symptoms may not be obvious. It also causes decreased distal temperatures, small-fiber neuropathy, and absent or reduced sensitivity to pain. Involvement of the brachial plexus, along with involvement of skin lesions, presents with marked hypersensitivity of its branches, trunk, and roots. As the disease progresses, sensitivity to heat changes, ranging from hyperesthesia to anesthesia. Compromised trunk neurons may present with paresthesias and muscle weakness. Although rare, central nervous system involvement may manifest as meningoencephalitis presenting with headache and neck stiffness [26,27,28,29,30]. The WHO suggests the use of a mixture of rifampicin, dapsone, and clofazimine to avoid drug resistance in the standard treatment for leprosy at both national and operational levels. Treatment for paucibacillary leprosy lasts six months, while multibacillary cases require 12 months. New neurologic dysfunctions require prompt anti-inflammatory treatment, typically with steroids for 4–6 months. Treatment is non-toxic in pregnancy, though pregnant women must be adequately treated as otherwise, the damage to the nerves may become irreversible [31,32,33,34,35,36]. The U.S. National Hansen’s Disease Program (NHDP) recommends a regimen that does not include clofazimine for treating paucibacillary leprosy and has a longer treatment period due to fewer economic constraints [37]. The NHDP suggests three alternative regimens: ofloxacin (400 mg) instead of clofazimine, minocycline (100 mg daily) instead of dapsone, and clarithromycin (500 mg daily) instead of any drug [38]. In addition, basic laboratory tests and liver function tests are recommended [39]. However, despite the indications of the WHO and the NHDP, there is a report of multidrug resistance to rifampicin and dapsone in Brazil, India, and Indonesia [40,41]. Finally, it is important to highlight that treatment effectiveness is directly linked to healthcare delivery and patient adherence. New techniques that address socioeconomic determinants of health are needed to increase patient adherence and promote adherence [42,43]. This scoping review aims to investigate the neurological manifestations and main treatments of leprosy patients. This objective is essential because the knowledge of the pathology of how leprosy interacts with the nervous system, especially with polyneuritis and mono neurosis, allows a clinician to anticipate and also treat neurological complications that are often the main cause of disability in patients with the disease. To better understand the topic, we summarized the neurological complications of leprosy in Figure 1.

## 2. Materials and Methods

### 2.1. Search Strategy

**Figure 2:**
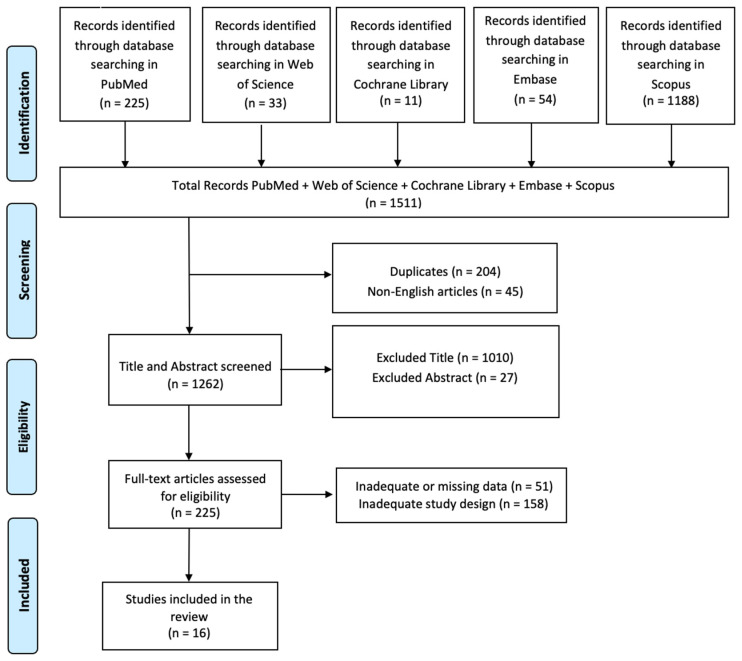
PRISMA 2020 flow diagram of evaluated studies.

A comprehensive literature search was performed using PubMed, Web of Science, Cochrane Library, Embase, and Scopus, employing the keywords (All Fields: “Leprosy”) AND (All Fields: “Neurological Complications”), without any time restrictions. The PRISMA (Preferred Reporting Items for Systematic Reviews and Meta-Analyses) flow diagram was utilized to outline the process (identification, screening, eligibility, and inclusion) for selecting relevant studies, as illustrated in Figure 2. Titles and abstracts from the database searches were independently reviewed. Articles were evaluated for their relevance based on predefined inclusion criteria. All titles and abstracts that met these criteria were fully reviewed. To minimize bias, multiple expert teams independently selected articles and analyzed data, discussing any discrepancies until consensus was achieved. This review has been registered on OSF with the number PQBYH.

### 2.2. PICO Evaluation

We utilized the PICO (population, intervention, comparison, outcome) model to establish our search terms. The population to feature in this review will include leprosy patients, especially those with neurological manifestations of neuritis, polyneuritis, and neuropathic pain. Its interventions involve a look into the various modes of treatments that make up MDT, with rifampicin, dapsone, clofazimine, and corticosteroids for neurological symptoms and reaction management. Comparison will involve treatments along different time courses depending on the type of drugs used, and also treatment regarding medical outcome versus surgical intervention such as nerve decompression. The outcome will be a reduction in neurological complications, improvement in nerve function, reduction in the rates of disability, and general recovery of the patient.

### 2.3. Inclusion Criteria

A study was included if it described or examined the neurological manifestations or rehabilitation treatments of leprosy patients. Only articles written in English were considered. Additionally, studies that described or investigated the functional assessment of these patients were included. We only included studies conducted in human populations and published in English that met the following criteria: (i) original or protocol studies of any kind and (ii) articles that detail the neurological manifestations or treatments of leprosy patients.

### 2.4. Exclusion Criteria

A study was excluded if it lacked data or information regarding the neurological manifestations or rehabilitation treatments of leprosy patients. Systematic, integrated, or narrative reviews were also excluded; however, their reference lists were reviewed and included when relevant. Additionally, any articles written in languages other than English were excluded.

## 3. Results

In total, 1511 articles were found: 204 articles were removed due to duplication after screening; 45 articles were excluded because they were not published in English; 1037 articles were excluded based on title and abstract screening. Finally, 209 articles were removed based on screening for inadequate study designs and untraceable articles (Figure 2). Sixteen research articles met the inclusion criteria and were, therefore, included in the review. These studies are summarized in Table 1.

The articles described in this review investigated the neurological manifestations and rehabilitation approaches of leprosy patients. The neurological complications and clinical factors were analyzed in six articles [44,45,46,47,48,49], the immunological reactions of leprosy in two papers [50,51], and the leprosy reactions in three research works [52,53,54]. The last five articles discussed the leprosy treatments [55,56,57,58,59].

**Table 1:** Summary of studies included in the research.

Author	Aim	Study Design/Intervention	Treatment Period	Sample Size	Outcomes Measures	Main Findings
Pitta et al. 2022 [44]	Describe the incidence of leprosy reactions in patients with PNL and their relationship to neuropathic pain	Retrospective Study	Between 1998 and 2016	52 patients with PNL and 67 with other clinical forms of leprosy	Dermatologist observations and nerve biopsy	PNL is a more immunologically stable form of leprosy, with more neuroinflammation than classical skin reactions and no association between acute neuritis and neuropathic pain
Reichart et al. 1982 [45]	To determine the incidence, prevalence, and nature of facial and trigeminal nerve lesions in relation to leprosy type, duration, and treatment	Retrospective Cohort Study	12 months	43 leprosy patients	BI, Clinical Examination	Facial nerve involvement in a disease typically occurs late, lasting 12.1 years, with the zygomatic branch being most affected and the maxillary branch being most affected by hypesthesia and anesthesia
Lasry-Levi et al. 2011 [46]	The study assesses the prevalence and clinical features of neuropathic pain in leprosy patients and the validity of the Douleur Neuropathique 4 questionnaire as a neuropathic pain screening tool	Cross-Sectional Study	Between July and August 2008	101 patients	Clinical neurological examination, assessment for leprosy.	Douleur Neuropathique 4 is highly sensitive and specific to the diagnosis of neuropathic pain, which is linked to nerve swelling, skin lesions, and psychological issues
Santos et al. 2015 [51]	To evaluate clinical factors associated with the development of disability in people with leprosy	Retrospective Study	Between 2001 and 2011	2358 cases	Assessment based on classification system of WHO	Being male, having two or more affected nerves, and being classified as multi-visceral leprosy, leprosy reaction, and lepromatous leprosy were found to be important factors associated with disability
Oliveira et al. 2013 [52]	To evaluate clinical factors associated with the development of leprosy reactions and physical disability in leprosy patients	Retrospective Study	From 2005 to 2011	494 patients	Scale of physical impairment according to WHO classification	At diagnosis, men were more likely to have multisclerotypic, reactive attacks and grade 2 physical disability. 40% of patients had a leprosy reaction, and all were treated with corticosteroids
Dell’Arco et al. 2016 [55]	To describe the challenges in the diagnosis and management of neuropathic pain caused by leprosy	Cross-Sectional Study	Not Specificated	85 patients	Douleur Neuropathic 4 test	Neuropathic pain in leprosy can be difficult to diagnose, and almost half of the patients surveyed were undiagnosed
Soysal et al. 2004 [56]	To define the types of peripheral neuropathy in leprosy patients	Retrospective Study	Between January–December 1999	29 patients	Medelec Sapphire 4ME EMG-EP device	The study found that sensory impairment was more severe in the lower limb than motor impairment, and no sympathetic cutaneous response was recorded in 79.3% of upper limb cases
Bandeira et al. 2019 [53]	To describe clinical and epidemiologic aspects of leprosy reactions in children in the Brazilian Amazon	Prospective Cohort Study	Between April 2014 and June 2015	34 leprosy patients	Structured Questionnaire	The study found that out of 34 patients, 52.9% had leprosy reactions and neuritis, with type I reactions occurring in 77.8% of cases and complications in 33.3%
Araujo et al. 2014 [47]	The study examines the neurological changes and disability in individuals with leprosy, focusing on their socio-demographic and clinical profile	Longitudinal Epidemiologic Study	Between March 2010 and February 2011	155 leprosy patients	The Brazilian Ministry of Health has developed a national protocol for simplified neurological assessment and classification of disability degree	Before treatment, 46.5% of patients had borderline skin, 51.6% eye and foot changes, and 18.7% radial nerve affected, with significant differences in changes before and after treatment
Shukla et al. 2020 [48]	A study on the correlation between ultrasonography and biopsy findings in leprosy patients with clinically diagnosed pure neuritis	Prospective Study	Not Specificated	100 patients with PNL	EHF	Ultrasonographic evaluation of peripheral nerves effectively detects thickening, facial structures, echogenicity, and vascularity, and nerve biopsy confirms leprosy bacteria in 75% of 32 cases
Soares et al. 2020 [49]	The study analyzed spatial luminance contrast sensitivity and color discrimination thresholds of protan, deutan, and tritan axes in leprosy patients	Cross-Sectional Study	Not Specificated	8 subjects with leprosy and 8 healthy subjects	Sociodemographic Questionnaire, Achromatic test, D15d, CCT	Leprosy affects visual processing of various spatial frequencies and cone sensitivity to short and long wavelengths, according to the study’s findings
Andrade et al. 2016 [50]	It was studied reactive leprosy patients with and without acute neuritis were studied	Cross-Sectional Study	Not Specificated	17 patients	Ridley-Jopling scale	ML may contribute to TNF-mediated inflammation and focal demyelination in the nerves of patients with leprosy neuropathy by making SCs more sensitive to TNF
Tiago et al. 2021 [57]	To evaluate the long-term (≥1 year) clinical and functional outcomes of PNSD in leprosy neuritis	Cross-Sectional Study	1-year	90 patients	SALSA scale	PNSD effectively reduces pain, improves motor function, and reduces long-term corticosteroid doses in leprosy patients, resulting in increased patient satisfaction
Jardim et al. 2007 [54]	This study will evaluate the efficacy of a combination of steroids and MDT in the prevention and arrest of nerve injury in PNL patients	Prospective Study	Between 1998 and 2000	24 PNL patients	VAS and electrophysiological examination	Full-dose PDN improved the clinical and electrophysiological status of patients with PNL and contributed to the prevention of further neurological damage
Anjayani et al. 2013 [58]	Effects of PRP to improve sensory innervation in leprosy patients	Randomized Controlled Trial	Not Specificated	60 subjects	TPDT, VAS	PRP injection into the perineurium of patients with leprosy peripheral neuropathy can improve peripheral nerve sensory function after 2 weeks of observation
Wagenaar et al. 2017 [59]	The study evaluates the effectiveness of a 32-week prednisolone course compared to a 20-week course in restoring and improving neurologic function in leprosy patients	Randomized Controlled Trial	February 2012 and October 2013, with the last follow-up data collected in July 2015	868 patients	RSS, SALSA, PS	Twenty weeks of prednisolone was as effective as 32 weeks in improving and restoring the final clinical NFI in leprosy patients

Legend: Reaction Severity Scale (RSS), Screening of Activity Limitation and Safety Awareness (SALSA), Participation Scale (PS), Nerve Function Impairment (NFI), Pure Neural Leprosy (PNL), multidrug therapy (MDT), Bacillary Index (BI), Nerve Growth Factor Receptor (NGFr), Protein Gene Product (PGP), World Health Organization (WHO), Eyes, Hand, Feet (EHF), Lanthony D-15 desaturated test (D15d), Cambridge color test (CCT), Mycobacterium leprae (ML), tumor necrosis factor (TNF), Schwann Cells (SCs), Peripheral Neural Surgical Decompression (PNSD), Visual Analogue Scales (VAS), Prednisone (PDN), Platelet-rich Plasma (PRP), Two-point Discrimination Test (TPDT), Visual Analog Scale (VAS).

### 3.1. Neurological Complications of Leprosy: A Clinical Perspective

Neurological symptoms and clinical characteristics play a crucial role in the diagnosis and rehabilitation treatment of leprosy. In a retrospective study involving 52 patients with pure neural leprosy (PNL) and 67 with other clinical forms of leprosy, it was observed that 23.1% of PNL patients experienced a neurological reaction during or after MDT, compared to 59.7% of patients with other forms of leprosy. There was an association between PNL and a lack of response during and after MDT, as well as post-MDT neuritis. Unlike other forms of leprosy, PNL was not linked to neuropathic pain, indicating that PNL is a more stable type of the disease. Understanding and identifying neuritis is essential for reducing disability and its public health impact [44]. A retrospective cohort study found diverse neurological symptoms among leprosy patients. Out of 43 individuals, 5 experienced partial mobility loss due to unilateral involvement of the right frontal branch, while the rest had a partial or total loss of movement. The zygomatic branch was solely damaged in 11 cases (3 partial, 8 total). Unilateral paralysis of the upper lip was observed in 10 cases (6 partial, 4 total), and unilateral loss of lower lip mobility occurred in 6 cases (4 partial, 2 total). In four cases, just one of the eight potential segments was affected, with the zygomatic area and Bell’s phenomenon leading to total loss of mobility. Trigeminal hypoesthesia and anesthesia were reported in 29 individuals, with the involvement of the frontal and zygomatic branches potentially due to the thin layer of soft tissue over the zygomatic bone and temperature drops linked to nerve injury. Early recovery from facial nerve palsy can lead to improper reinnervation, causing voluntary contractions of unrelated muscles [45]. In a cross-sectional study of 1001 patients, 22 (21.8%) had neuropathic pain. The main sensory symptoms included numbness (86.4%), tingling (68.2%), decreased tactile sensation (81.2%), and pinpoint irritation (72.7%). Neuropathic pain was associated with nerve swelling, tenderness, painful skin lesions, and psychological issues. The Douleur Neuropathique 4 had a sensitivity and specificity of 100% and 92%, respectively, for diagnosing neuropathic pain [46]. An epidemiological study of 153 leprosy patients showed that 46.5% were borderline before MDT, with significant changes observed in the eyes, feet, and radial nerves. Hand complications improved after treatment, but vascular complications remained high. Neurological complications also showed varied improvement, with some nerve areas worsening post-treatment (radial nerve, median nerve, and peroneal nerve) [47]. A prospective study discovered that polyneuritis/mononeuritis was present in 75% of cases, with mononeuropathy in 18% and polyneuritis in 7%. Ultrasonographic evaluation of peripheral nerves was superior to clinical examination for detecting thickening and characterizing facial structures. A nerve biopsy confirmed leprosy in 75% of cases, with cranial nerve lesions and bilateral thickening of the great auricular nerve significantly associated with leprosy bacteria positivity. Disability scores improved significantly after MDT [48]. In the study of Soares et al., neurological complications were much more serious in patients with leprosy when compared with healthy controls. All had normal visual acuity, but the contrast sensitivity was notably lower at various spatial frequencies among the subjects with leprosy. The color confusion index, which reflects higher difficulty with color discrimination, was greater in leprosy patients. Notable variances were also observed in the tritan confusion axis. Sizes of ellipses from color discrimination tests were larger in leprosy patients, reflecting greater color confusion, which did not correlate with either treatment time or age. Overall results suggest that leprosy can result in significant deficits in both contrast sensitivity and color discrimination as a part of broader neuropathological complications [49]. Recognizing and addressing these neurological symptoms is vital for improving patient outcomes and mitigating the long-term impacts of leprosy on individuals’ health and quality of life.

### 3.2. Immunological Reactions in Leprosy: Clinical Implications and Nerve Damage

This section investigates the immunological reactions in leprosy patients, focusing on their clinical manifestations and correlation with peripheral nerve damage. A cross-sectional study of nerve conduction in leprosy reaction patients demonstrated demyelination in all of the patients with acute neuritis, though there was no correlation between antibodies against gangliosides and clinical demyelination. Tumor necrosis factor (TNF), TNF receptors, and TNF-converting enzymes were detected in Schwann cells by immunofluorescence in nerve biopsies. Infection of Schwann cells by Mycobacterium leprae further stimulated the expression of transmembrane TNF and TNF receptor 1. Moreover, TNF had an inducing effect on interleukin-6 and interleukin-8 secretion, whereas Mycobacterium leprae induced interleukin-23 release. They concluded that Mycobacterium leprae enhanced the sensitivity of Schwann cells to TNF, promoting TNF-mediated inflammation and focal demyelination, thus contributing to nerve damage in leprous neuropathy [50]. A retrospective study tried to find clinical factors that predispose physical disabilities in cases of leprosy, focusing on the immune reactions involved. This research article thus analyzed 2358 leprosy cases in Aracaju, Brazil, from 2001 to 2011 and found the important associations with physical disability, which included being of male gender, having more than two affected nerves, and multibacillary leprosy classification. Leprosy reactions, as an indicator of immune responses, were significantly associated with increased disability. The multibacillary form of the disease had an adjusted odds ratio of 2.74, and importantly, lepromatous leprosy had an adjusted odds ratio of 4.87. These results bring out at this stage that the immune responses reflecting the severity of infection, especially in reactional states, and the extent of nerve involvement are important predictors of the development of disability [51]. These results implicate the immune response at the center of disease manifestations in leprosy, serving not only as a marker of loss but also as a potential target for therapeutic intervention aimed at the prevention of nerve damage.

### 3.3. Leprosy Reactions in Patients: Challenges and Improvements

This section examines the prevalence and consequences of leprosy reactions among patients, highlighting their association with corticosteroid treatment and physical impairment. In a retrospective study, 40% of the patients had leprosy reactions, and all of them received corticosteroid treatment. Male patients were more significantly affected by multibacillary forms and reactional episodes that led to grade 2 physical impairment at the time of diagnosis. At the end of the treatment, 29.8% of the patients still presented physical impairment. Multivariate analysis demonstrated that these impairments persisted when a low dose and short duration of corticosteroid treatment were used [52]. A prospective cohort study in children with leprosy found that 52.9% of the 34 patients had leprosy reactions and/or neuritis. One-third of patients experienced complications such as erythema nodosum, necrotizing erythema, or Cushing’s syndrome. Age, the number of doctors seen, polymicrobial classification, skin lesions, and borderline clinical forms were significantly associated with leprosy reactions. The prevalence of type I reactions indicated a need for prolonged corticosteroid treatment, potentially affecting bone development in children [53], which started within 2 to 120 months. The median time was 14 months. A shocking finding during examination is that 75% of them had developed disability grade 2, including such deformities as ulcers and claw fingers/toes. On sensory assessment, median nerve impairment was found in 42%, while on motor assessment, the commonest was ulnar nerve dysfunction, found in 38%. Only 8% of the patients developed acute neuritis after MDT during follow-up. Although some of them had maintained sensory impairment, the sensory scores significantly improved in 71% of them. Muscle strength had improved in 63% of patients, while none showed worsening. The affected nerves were reduced though demyelinating lesions persisted, thus suggesting that leprosy reactions may result in significant clinical improvements after treatment; 94.7% of the patients were able to conclude the treatment with at least one normal nerve [54]. These findings emphasize the importance of tailored corticosteroid therapies and the need for ongoing monitoring to manage leprosy reactions effectively and minimize long-term disabilities.

### 3.4. Leprosy Treatments: Insights into Pain and Neurological Function

This section presents the findings of various studies focused on the treatment and management of leprosy patients, particularly regarding pain and neurological complications. In a cross-sectional study, a total of 85 leprosy patients were studied, and 37 were in a state of either nociceptive or neuropathic pain. Out of these, 22 fulfilled the criteria for neuropathic pain. Of these patients, 63.7% were women, and 68.1% had multibacillary leprosy; the majority knew their diagnosis for over five years. Diagnosis errors occurred in 45.5% of the cases, the main ones by dermatologists. All 12 patients with neuropathic pain were on tricyclic antidepressants, usually amitriptyline, although two were dropped because of side effects. Treatment failures also could be attributed to inadequate dosing and the absence of combination therapy. Patients who were treated with either gabapentin or pregabalin experienced some relief, and a comparison of pain control between the treated and untreated groups yielded an overall significant difference in the means of the pain scores for those treated vs. untreated [55]. In a retrospective study of 30 leprosy patients with a mean disease duration of 33 years, 21 were inactive, and 3 were relapsing. The major diagnoses were lepromatous leprosy in 19 and borderline lepromatous leprosy in 10. Nerve thickening was widespread, and the sensory loss was asymmetrical, but deep tendon reflexes were preserved in all. Of the 23 patients who had MDT, all were drug-free for 2–17 years. Electrophysiological studies revealed the absence of motor response in 42 peroneal and 37 tibial nerves; sensory nerve action potentials could be recorded in only 19 medians and 9 ulnar nerves. Overall, lepromatous leprosy patients exhibited more severe nerve involvement than those with borderline lepromatous leprosy, highlighting the significant impact of the disease on both motor and sensory nerves [56]. A cross-sectional study found that surgical decompression of peripheral nerves in leprosy effectively reduced pain prevalence and intensity, improved motor function, and decreased corticosteroid doses, as reflected in patient satisfaction. Of the 246 nerves operated on in 90 patients, most surgeries involved decompression of the median and ulnar nerves. Approximately 77% of patients (69/90 patients) underwent surgery on only one limb (two nerves), 14.4% (13/90 patients) on two limbs (four nerves), 4.4% (4/90 patients) on three limbs (six nerves), and 4.4% (4/90 patients) on four limbs (eight nerves) [57]. In a randomized controlled trial, perineural injections of platelet-rich plasma outperformed worse plasma in improving scores on the visual analog scale and two-point discrimination test. Both groups reported feeling the injection, but the tingling sensation subsided immediately. Within two weeks of monitoring, injecting platelet-rich plasma into the peripheral nerves of leprosy patients enhanced sensory function [58]. In another study, 868 leprosy patients with recent neurological impairment (less than six months) were randomly assigned to either a 20-week (429 patients) or a 32-week (439 patients) prednisolone therapy regimen. Prednisolone was administered at 45 mg or 60 mg per day, depending on the patient’s weight, and gradually tapered. By week 78, 78.1% of patients in the 20-week group and 77.5% in the 32-week group showed improved or regained neurological function, with no significant difference (*p* = 0.821). Secondary outcomes were similar between the groups, except for a significantly higher risk of major adverse events in the extended treatment group. Consequently, an initial treatment duration of 20 weeks for leprosy neuropathy is recommended, with relatively few patients needing additional tailored therapy [59]. Overall, these results underscore the complexity of treating leprosy-related neuropathy and the importance of individualized treatment strategies to improve patient outcomes.

## 4. Discussion

This scoping review aimed to analyze the neurological manifestations and rehabilitation treatments of leprosy patients. These recent studies on neurologic involvement in leprosy throw light on various important features of manifestations, modes of treatment, and laboratory diagnostic difficulties, all of which form a more varied concept about the condition. The main attack of leprosy is on the peripheral nervous system, which results in various neurological complications [60,61]. One research study found that 23.1% of patients with PNL experienced a leprosy reaction either during or after MDT treatment. In contrast, this jumped considerably to 59.7% in patients with other clinical forms of leprosy [62]. The stability of PNL, when compared with the other forms, signals an important difference concerning treatment response and prognosis. Neurological symptoms, including partial loss of mobility and unilateral facial paralysis, also underscore the fact that early recognition and management of such complications would result in averting disability and public health consequences [63]. Additionally, a sizeable proportion of leprosy patients are suffering from neuropathic pain, and various reports point to sensory symptoms such as numbness and tingling [64]. These symptoms are indicative of nerve swelling and psychological problems, thus pinpointing the interrelation between somatic and psychic entities in leprosy patients. The strong prevalence of neurological symptoms calls for routine neurological examinations and broad rehabilitation strategies to improve the quality of life. All experts agree that immunological reactions in leprosy play a crucial role in nerve damage [65]. Several studies confirm that leprosy can lead to gross peripheral neuritis and that there is a very alarming correlation between immunological reactions and the occurrence of physical disabilities [66]. Indeed, male gender, extensive nerve involvement, and multibacillary classification have been established as strong predictors of disability. These findings are in concert with the literature that underlines the position of immune responses in determining the clinical manifestations of leprosy and offers potential ways for therapeutic intervention targeted at protecting nerve function. Leprosy reactions are debilitating to manage, and because almost 40% of patients do experience such reactions, corticosteroid treatment so often has to be used to prevent physical impairment [67]. Nevertheless, the efficacy of corticosteroid therapy significantly relies on the dosage and length of treatment. Lower doses for brief durations are generally more successful in managing symptoms with minimal side effects, whereas extended use or elevated doses may result in negative effects. These encompass heightened vulnerability to infections, weight increase, elevated blood sugar, brittle bones, and digestive problems. Prolonged use of corticosteroids may cause hormonal imbalances, resulting in mood disturbances like anxiety and depression, especially in susceptible groups like children. Furthermore, there are worries regarding growth suppression in children and the potential for metabolic issues, highlighting the importance of closely monitoring patients throughout treatment. Considering these possible side effects, corticosteroid treatment should be customized to each patient’s requirements, with close attention to the risks and advantages [68,69]. Furthermore, it is important to note that MDT can successfully eliminate Mycobacterium leprae but does not completely address the neurological complications arising from the infection. Peripheral nerve degeneration continues primarily because of leprosy’s effects on the peripheral nervous system, leading to ongoing nerve fiber damage from direct bacterial invasion and immune-mediated inflammatory reactions. Despite decreasing the bacterial load, leprosy reactions, such as type 1 and type 2, can worsen inflammation during or after MDT. This persistent inflammation can result in permanent nerve injury, showing up as sensory deficits, motor issues, and autonomic irregularities. Furthermore, the mechanisms for nerve repair are restricted since, after bacterial clearance, injured nerves frequently have difficulty regenerating, especially when there is accompanying scarring. Management of leprosy neuropathy is multidimensional and includes both pharmacologic and surgical measures. Indeed, various studies have documented that the mainstay of treatment remains tricyclic antidepressants; among them, amitriptyline is commonly prescribed [70]. Most treatment failures are due to misdiagnosis and improper dosing. The small benefit reported with gabapentin or pregabalin suggests an investigation into polypharmacotherapy that could further lead to adequate pain relief [71]. The application of selective serotonin-–norepinephrine reuptake inhibitors (SNRIs), like duloxetine, has demonstrated potential in treating neuropathic pain, offering fewer side effects than tricyclic antidepressants. Additionally, the possibility of employing anti-inflammatory medications like methotrexate or biological agents aimed at specific immune responses is being investigated to address leprosy reactions and avert nerve harm. Recent studies have also explored the function of antioxidants and neuroprotective substances, such as N-acetylcysteine, which might assist in reducing oxidative stress and promoting nerve regeneration. These options might enhance current MDT treatments by targeting both the bacterial infection and the inflammatory mechanisms causing nerve injury [72,73,74,75,76]. Surgical interventions, such as peripheral nerve decompression, have also emerged as effective modalities of pain relief and restoration of motor functions. This is supported by the fact that these surgical approaches significantly enhance patient satisfaction and reduce dependency on corticosteroids. Other novel treatments, such as the perineural injections of platelet-rich plasma, seem promising in terms of improving sensory function and need further investigation [77]. Recent trials indicate that a 20-week course of prednisolone is beneficial in leprosy neuropathy, with significant improvements in neurological function [78]. This again speaks to the fact that treatments need to be personalized since there is great variability in response among patients, which requires elasticity in therapeutic intervention. Besides the above-mentioned aspects of leprosy, it is relevant to discuss the issue of subclinical neuropathy, often unnoticed and yet so vital in the overall management and understanding of the disease. Subclinical neuropathy is defined as damage to the nerves without overt manifestations; however, this can be identified by clinical or diagnostic investigations [79]. Recent studies emphasize that a significant proportion of leprosy patients may have evidence of subclinical neuropathy, often serving as a precursor to the development of overt neurological manifestations. This further necessitates an early detection approach and proactive management. The fact that this neuropathy may also be subclinical is quite worrisome because it predisposes one to further damage, hence increased symptoms and disability. Patients with subclinical neuropathy can have preventive measures focused on them, which include a more intensive follow-up and tailored interventions that reduce the risk of progression into symptomatic neuropathy [80]. The detection of subclinical neuropathy has the potential to greatly improve clinical outcomes in leprosy patients. Standardized testing, including nerve conduction studies and sensory testing, is important in ascertaining patients in whom clinically significant nerve damage is likely to occur. Such patients can be treated with early intervention measures, which include educating them on self-care practices, among others, with regular follow-up. Moreover, the identification of subclinical neuropathy raises the importance of a holistic approach to the care of leprosy patients by including regular neurological evaluation in follow-up care [81]. This can allow timely implementation of effective interventions to avoid irreversible nerve damage and improve functional outcomes and quality of life in patients. The integration of the concept of subclinical neuropathy into clinical practice requires a paradigm shift in the approach to the management of leprosy. Healthcare providers must be trained regarding the recognition of signs and symptoms of subclinical neuropathy in cases without overt clinical manifestations [82]. Standardized approaches for nerve function assessment can provide an early detection of impairments, which allows timely interventions. Apart from the neurological impact of leprosy itself and the treatment strategies, major challenges in laboratory diagnostics play a significant role in the management of patients. These include the lack of sensitive and specific diagnostic tests leading to a large proportion of misdiagnosis or delayed diagnosis [83]. Slit-skin smears and histopathological examination, among others, can give false negatives, especially in the case of early or subclinical leprosy. Molecular techniques, such as polymerase chain reaction, hold promise for much better sensitivity but are not widely available in resource-poor settings. Serological tests targeting antibodies against Mycobacterium leprae have their drawbacks due to variability in immune responses among patients. The various methodologies that are in place create a harsh reality for the timely identification of leprosy and may thus allow neurological damage to progress before treatment can begin. Therefore, investment in developing reliable and accessible diagnostic methods that identify leprosy, even its subclinical forms, should be performed so effective early intervention can be pursued for an improved status of the patients. Alongside enhancing diagnostic abilities, public health efforts are vital in decreasing the worldwide impact of leprosy. Minimizing the stigma linked to the illness is crucial to motivating those impacted to pursue prompt medical attention. Leprosy is frequently misconceived, and numerous patients postpone treatment due to anxiety about discrimination or social isolation. By increasing awareness of the illness and its therapies, public health initiatives can diminish misunderstandings and create an atmosphere where patients feel secure seeking medical care. Regular neurological assessments are crucial in the care of leprosy patients since neurological issues are frequent but frequently overlooked. Numerous people affected by leprosy go through subclinical neuropathy, a state where nerve harm happens without evident signs. This quiet advancement of nerve injury can ultimately result in lasting disability if not recognized and treated promptly. Consistent neurological evaluations, such as nerve conduction tests and sensory assessments, can aid in identifying subclinical neuropathy and stop its progression to more serious types of nerve injury. Furthermore, it is crucial to perform regular follow-up care for leprosy patients, especially for those susceptible to developing symptomatic neuropathy. Preventive strategies, such as educating patients on self-care and appropriate limb protection, are essential for reducing the likelihood of complications. An extensive, multidisciplinary strategy that includes consistent monitoring, prompt interventions, and patient education can greatly decrease the occurrence of disability and enhance the overall quality of life for those impacted by leprosy [84,85,86,87,88].

This scoping review has several strengths. It is grounded in evidence from studies analyzing neurological complications specific to leprosy patients and includes descriptions of the rehabilitation treatments for this condition. The review is based on both retrospective and prospective studies with large samples. However, the main limitation of this study is the limited number of articles that met the inclusion criteria, with only seventeen articles exploring neurological complications and clinical aspects in leprosy patients. This, combined with methodological variations and heterogeneous samples, hinders the ability to draw solid conclusions on this important topic. Additionally, the sample sizes varied; some were large, others were small, and the parameters measured differed. While the pharmacological and rehabilitative treatments analyzed are still under study to reduce the neurological and clinical symptoms caused by leprosy, the initial results are promising. Further clinical trials are needed to identify specific drugs and rehabilitation therapies based on robust scientific evidence. The review also highlighted the limitations of the screening and tracking tools for this condition, which are crucial for preventive intervention and treatment.

## 5. Conclusions

In conclusion, neurological complications of leprosy require much care in diagnosis and treatment by pharmacological and surgical means. This suggests that even with the advent of MDT, many patients still present with peripheral degenerative neuropathy, which may not heal properly and, therefore, require further explorations into the pathophysiological background. Indeed, future studies on the validation of classification methods, early diagnostic tools, and the role of anti-inflammatory treatments, including steroids, will be critical in the management of the disease. Further, the relationship between neuropathic pain and psychological morbidity can also be explored to add to the interventions that may improve patients’ quality of life. Needless to say, effective vaccines and rapid diagnostic tests for Mycobacterium leprae will hasten the day when infection with this bacillus no longer menaces people with this illness. With emerging diagnostics, new pharmacological interventions, and an increase in sophistication about disease mechanisms, the future of leprosy management looks bright, with hope for better outcomes among the affected individuals and mitigation of neurological complication burdens.

## Data Availability

Not applicable.
